# Abstract of Meteorological Observations for January, 1856, Made at Philadelphia, Pa.

**Published:** 1856-03

**Authors:** James A. Kirkpatrick


*Abstract of Meteorological Observations for January,* 1856, *made at Philadelphia, Pa. Latitude* 39° 57′ 28″ *N., Longitude* 75° 10″ 40′ *W. from Greenwich.* By PROF. JAMES A. KIRKPATRICK.

| 1856. January. | Barometer. Daily Mean | Barometer. Mean Daily Range. | Thermom. Daily Mean | Thermom. Mean Daily Range | Rel. Humid. 2 P.M. | Force of Vapor 2 P.M. | Dew Point 2 P.M. | Rain & Melted Snow. | Prevailing Winds. | Remarks. |
|---|---|---|---|---|---|---|---|---|---|---|
| | Inches. | Inches. | Deg. | Deg. | Hunds. | Inches. | Deg. | Inches. | Points. | |
| 1 | 30.148 | .102 | 25.7 | 3.8 | 67 | .112 | 20.8 | | NW. | M. and ev. clear; aft. cl'dy. |
| 2 | 30.165 | .155 | 28.0 | 2.3 | 79 | ·143 | 26.4 | 0.884 (days 2–3) | NE. | Cl'y; ev. *sleet*, changing at 9½ P. M. to *rain*. |
| 3 | 29.782 | .383 | 35.7 | 7.7 | 90 | .205 | 35.1 | | NW. | M. & aft. cl'dy; m. *rain*, 10 A. M. to 12 *fog*, ev. clear. |
| 4 | 30.328 | .546 | 21.0 | 14.7 | 50 | .067 | 9.6 | | (Var.) | Cl'r; *Bar. highest* 30.381 in. |
| 5 | 29.977 | .351 | 18.2 | 4.8 | 100 | .096 | 17.4 | 0.533 | NE. | Cl'y; 1½ P. M. *snow*; stopped during the night. about |
| 6 | 29.927 | .288 | 23.3 | 6.8 | 71 | .112 | 20.8 | | W. | Clear [18 inches deep. |
| 7 | 29.971 | .151 | 19.0 | 6.0 | 75 | .100 | 18.3 | | WNW. | Cloudy; m. *fog*. |
| 8 | 29.812 | .171 | 18.7 | 11.0 | 49 | .089 | 15.8 | | (Var.) | Cloudy: m. *fog*. |
| 9 | 30.034 | .223 | –1.0 | 19.7 | 72 | .034 | –5.3 | | WNW. | Clear; *Thermom. lowest* 5° |
| 10 | 30.100 | .066 | 6.7 | 7.7 | 61 | .046 | 1.3 | | W. | Clear. [*below zero*. |
| 11 | 30.253 | .153 | 18.3 | 11.7 | 62 | .083 | 14.3 | | W. | Clear. |
| 12 | 30.066 | .190 | 24.0 | 6.3 | 87 | .112 | 20.8 | 1.410 (days 12–13) | NE. | Cl'y; 5 P. M. *snow* till 10 P. M., then heavy *rain* with high wind. |
| 13 | 29.309 | .757 | 34.5 | 10.5 | 79 | .162 | 29.3 | | (Var.) | Cl'y, *rain* till 8 A. M., 1 P. M. snow for half an hour. *Bar. lowest* 29.266 in |
| 14 | 29.624 | .315 | 31.0 | 3.5 | 61 | ·120 | 22.4 | | W. | M. cl'r; aft & ev. cl'y, light *snow* from 3 to 4 P. M, |
| 15 | 29.694 | .069 | 27.5 | 3.5 | 66 | .106 | 19.6 | | WNW. | M. clear, aft. and ev. cl'dy. |
| 16 | 29.631 | .065 | 30.3 | 4.0 | 72 | .146 | 26.9 | 0·018 | (Var.) | M. & aft. cl'y, ev. cl'r, *snow* during night. |
| 17 | 29.577 | .122 | 30.3 | 1.3 | 70 | .132 | 24.6 | | W. | M. aft. & cl'dy, ev. clear. |
| 18 | 29.723 | .147 | 33.5 | 4.5 | 64 | .152 | 27.8 | | W. | Clear. |
| 19 | 29.70 | .055 | 33.0 | 2.5 | 58 | .155 | 28.3 | | NW. | Cloudy. *Ther. highest* 42° |
| 20 | 29.671 | .077 | 17.3 | 11.7 | 84 | .083 | 14.3 | 0.006 | NE. | Cl'y, *snow* 9 A. M. to 2 P. M. |
| 21 | 29.685 | .038 | 18.7 | 2.0 | 57 | .064 | 8.6 | | NW. | M. & aft. cl'y, light snow at |
| 22 | 29.827 | .142 | 21.0 | 3.0 | 62 | .083 | 14.3 | | W. | Clear. [7½ A. M., ev. clear. |
| 23 | 29.903 | .076 | 25.3 | 4.3 | 77 | .124 | 23.2 | | W. | Aft cl'dy, m. & ev. clear. |
| 24 | 29.921 | .045 | 25.3 | 2.7 | 57 | .095 | 17.2 | | (Var.) | M. & aft. clear, ev. cloudy. |
| 25 | 30.219 | .298 | 15.7 | 9.7 | 68 | .067 | 9.6 | | WNW. | M. cl'y, aft. & ev. clear. |
| 26 | 30.269 | .149 | 19.5 | 8.5 | 62 | .083 | 14.3 | | WNW. | Clear. |
| 27 | 29.782 | .487 | 23.8 | 5.3 | 93 | .118 | 22.0 | 0.238 | NE. | Cloudy; m. & aft. *snow*. |
| 28 | 29.517 | .317 | 29.2 | 6.2 | 79 | .136 | 25.2 | 0·195 | (Var.] | Cl'y; m. & aft. *snow* about |
| 29 | 29.767 | .250 | 32.0 | 2.8 | 76 | .163 | 29.5 | | W. | Cl'dy. [5 in. in the 2 days. |
| 30 | 29.725 | .084 | 24.7 | 7.3 | 48 | .083 | 14·3 | | W. | Clear. [ped during night. |
| 31 | 29.878 | .152 | 21.0 | 5.7 | 63 | .086 | 15.1 | 0.084 | [Var.] | M. clear, 7½ P. M. *snow*, stop- |
| Means for Jan., 1856 | 29.870 | .207 | 23.6 | 6.5 | 70 | .103 | 18.8 | 3.368 | N. 55° 50′ W. 73-100. | |
| Means for Jan., 5 yrs | 29.941 | .206 | 29.3 | 6.6 | 74 | .150 | 27.1 | 2.428 | N. 56° 7′ W 55-100. | |

The Monthly Range of the Mercury in the Barometer was 1.115 inches, and in the Thermometer 47°.

